# Magnetic resonance imaging-based changes in vascular morphology and cerebral perfusion in subacute ischemic stroke

**DOI:** 10.1177/0271678X211010071

**Published:** 2021-04-17

**Authors:** Anna Kufner, Ahmed A Khalil, Ivana Galinovic, Elias Kellner, Ralf Mekle, Torsten Rackoll, Philipp Boehm-Sturm, Jochen B Fiebach, Agnes Flöel, Martin Ebinger, Matthias Endres, Alexander H Nave

**Affiliations:** 1 Charité–Universitätsmedizin Berlin, corporate member of Freie Universität Berlin and Humboldt-Universität zu Berlin, Center for Stroke Research Berlin, Berlin, Germany; 2 Klinik und Hochschulambulanz für Neurologie, Charité – Universitätsmedizin Berlin, Berlin, Germany; 3 Berlin Institute of Health, Berlin, Germany; 4 School of Mind and Brain, Humboldt Universität zu Berlin, Berlin, Germany; 5 Department of Neurology, Max Plank Institute for Human Cognitive and Brain Sciences, Leipzig, Germany; 6 Department of Radiology, Medical Physics, University Medical Center Freiburg, Freiburg, Germany; 7 QUEST Center for Transforming Biomedical Research, Berlin Institute of Health, Berlin, Germany; 8 ExcellenceCluster NeuroCure, Charite-Universitätsmedizin Berlin, Berlin, Germany; 9 NeuroCure Cluster of Excellence and Charité Core Facility 7T Experimental MRIs, Charité – Universitätsmedizin Berlin, Berlin, Germany; 10 Department of Experimental Neurology, Charité-Universitätsmedizin Berlin, Berlin, Germany; 11 Department of Neurology, University Medicine Greifswald, Greifswald, Germany; 12 German Center for Neurodegenerative Diseases, Partner Site Rostock/Greifswald, Greifswald, Germany; 13 Department of Neurology, Medical Park Berlin Humboldtmühle, Berlin, Germany; 14 German Centre for Cardiovascular Research (DZHK), Berlin, Germany; 15 German Center for Neurodegenerative Diseases (DZNE), Berlin, Germany

**Keywords:** Angiogenesis, ischemic stroke, microvasculature, perfusion, vessel size imaging

## Abstract

MRI-based vessel size imaging (VSI) allows for *in-vivo* assessment of cerebral microvasculature and perfusion. This exploratory analysis of vessel size (VS) and density (Q; both assessed via VSI) in the subacute phase of ischemic stroke involved sixty-two patients from the BAPTISe cohort (‘Biomarkers And Perfusion--Training-Induced changes after Stroke’) nested within a randomized controlled trial (intervention: 4-week training *vs.* relaxation). Relative VS, Q, cerebral blood volume (rCBV) and –flow (rCBF) were calculated for: ischemic lesion, perilesional tissue, and region corresponding to ischemic lesion on the contralateral side (mirrored lesion). Linear mixed-models detected significantly increased rVS and decreased rQ within the ischemic lesion compared to the mirrored lesion (coefficient[standard error]: 0.2[0.08] p = 0.03 and −1.0[0.3] p = 0.02, respectively); lesion rCBF and rCBV were also significantly reduced. Mixed-models did not identify time-to-MRI, nor training as modifying factors in terms of rVS or rQ up to two months post-stroke. Larger lesion VS was associated with larger lesion volumes (β 34, 95%CI 6.2–62; p = 0.02) and higher baseline NIHSS (β 3.0, 95%CI 0.49–5.3;p = 0.02), but was not predictive of six-month outcome. In summary, VSI can assess the cerebral microvasculature and tissue perfusion in the subacute phases of ischemic stroke, and may carry relevant prognostic value in terms of lesion volume and stroke severity.

## Introduction

Vessel size imaging (VSI) is a magnetic resonance imaging (MRI) sequence that has emerged as a promising technique able to non-invasively assess microvasculature morphology and cerebral perfusion in rodents and humans.^1–3^ VSI is based on a multi-contrast MR-acquisition that includes using a contrast agent, which leads to susceptibility changes in the vasculature, which are influenced by morphological properties of the vascular network. By means of analytical modeling, this method allows for the *in-vivo* assessment of the vascular density (reflected by Q), average vessel size (VS) in µm, and relative tissue perfusion.^1^

Previous studies in rat stroke models have shown that quantitative measurements derived from VSI highly correlate with the observed histological changes in vessel morphologies.^4,5^ Subsequent studies in humans - including ischemic stroke patients - confirmed that following an acute ischemic stroke there is an increase in the mean VS and decrease in vessel density within the ischemic lesion.^6,7^ In the acute phase following ischemia, an increase in lesion VS is likely a reflection of vessel dilation in a presumed attempt to increase tissue perfusion; an alternative hypothesis suggests that the compression of small capillaries following focal cytotoxic edema leads to an overall shift in calculated average VS to larger values.^7^ In subacute phases of stroke, there is discussion whether VSI may aid in the assessment of vascular remodeling in the context of angiogenesis; the formation of abnormal, bulky vessels in post-ischemic areas may lead to increased lesion VS measurements.^8^ Although only a handful of preclinical studies have applied VSI to evaluate vascular remodeling in subacute stages of ischemia,^9,10^ clinical VSI analyses in subacute and chronic stages following stroke in patients are still lacking.

Angiogenesis and vascular remodeling following ischemic stroke play a crucial role in recovery of damaged tissue and post-stroke functional recovery.^11,12^ In-vivo visualization of early vascular remodeling may aid in identifying those patients most likely to regain function post-stroke,^13^ if a clear clinical benefit of angiogenesis is observed in patients in future studies. Although pre-clinical murine stroke studies have well characterized the complex physiological cascade leading to changes in the neighboring vascular bed following ischemia,^4,12–15^ studies investigating potential angiogenesis in ischemic stroke patients are still generally lacking.^11^ The use of VSI may allow for the in-vivo assessment of ischemia-induced changes in the microvasculature in subacute ischemic stroke patients and could help us to better understand vascular remodeling and functional recovery post-stroke.

### Aim

This is a pre-planned, exploratory analysis of ischemic stroke patients with prospectively acquired MRI data as part of the observational BAPTISe study (‘Biomarkers And Perfusion – Training-Induced changes after Stroke’, clinicaltrials.gov identifier: NCT NCT01954797), which aimed to study potential markers (including MRI parameters) of angiogenesis.^16^ All patients were enrolled in the randomized, controlled trial PHYS-STROKE (‘Physical Fitness Training in Sub-acute Stroke’ clinicaltrials.gov identifier: NCT NCT01953549).^17,18^

The primary aim of the current study was to investigate vessel size and density (assessed via VSI) and cerebral perfusion (assessed via VSI or dynamic susceptibility contrast perfusion weighted-imaging [DCS-PWI]) in the subacute phase following an ischemic stroke in selected regions of interest, including the ischemic lesion and perilesional space. Furthermore, our aim was to assess whether vessel size within the ischemic lesion is associated with time from stroke onset to imaging and stroke progression (i.e. stroke severity, lesion size, and functional recovery at six months after stroke).

## Materials and methods

### Data availability statement

Raw imaging data are not publicly available as they contain information that could compromise patient privacy; program code of the imaging processing pipeline can be shared upon request. Numerical data has been uploaded onto an open repository and can be accessed via the following link: https://doi.org/10.6084/m9.figshare.13176647.v1.

### Patients and study design

All patients participated in the BAPTISe study nested within the randomized controlled PHYS-STROKE trial; a detailed description of all inclusion and exclusion criteria of PHYS-STROKE and BAPTISe are listed in the Supplemental files. All participants provided informed consent. The study was approved by the institutional review board of Charité–Universitätsmedizin Berlin (EA1/138/13). The original PHYS-STROKE trial was conducted in line with the CONSORT extension for non-drug treatments, and study procedures were carried out in accordance with the Declaration of Helsinki.

Patients enrolled in PHYS-STROKE were randomized to four weeks of aerobic physical fitness training or four weeks of relaxation courses (in addition to standard rehabilitation) and patients were eligible to participate in BAPTISe if they experienced an ischemic stroke and had no MRI contraindications. In BAPTISe, all patients received an MRI before (v01) and after (v02) the intervention. Patients received a clinical follow-up for six months after stroke; for the current analysis of functional outcome we included the assessment of stroke severity via the National Institute of Health Stroke Scale (NIHSS; at time of stroke onset, v01, and v02) and functional recovery via the modified Ranking Scale (mRS; at six months post-stroke). Detailed study protocols were published previously.^12,17^ For the current analysis, patients had to receive at least one MRI either before or after intervention, which included either VSI and/or DSC-PWI. Only patients with non-lacunar supratentorial strokes were included in this analysis to allow for accurate VSI analysis of the ischemic lesion.

### Imaging

All studies were performed using a 3-Tesla clinical MRI scanner (Tim Trio, Siemens Healthineers, Erlangen, Germany). The MR protocol included a FLAIR sequence (TE = 100 ms, TR = 8000 ms, TI = 2370.5 ms, field-of view [FOV] = 220 mm^2^, matrix = 256 × 232, 5-mm slice thickness with a 0.5-mm interslice gap) and diffusion-weighted imaging (echo time [TE] = 93.1 ms, repetition time [TR] = 7600 ms, 6 spatial directions, b = 0 and 1000 s/mm^2^, FOV = 230 mm^2^, matrix = 192 × 192, 2.5-mm slice thickness with no interslice gap, 25 slices).^16^

In addition, VSI measurements were acquired using a combined spin-echo (SE) and gradient-echo (GRE) single-shot 2 D Echo Planar Imaging (EPI) sequence (TEGRE = 22 ms, TESE = 85 ms, TR = 1890 ms, Flip Angle = 90°, FOV = 230 mm, matrix size 64×64, 16 slices, slice thickness = 5 mm, no gap, Bandwidth = 2111 Hz/Pixel, 60 repetitions). A total of 0.13 mL/kg body weight of contrast agent (Gadolinium) was injected intravenously (5 mL/second) 5 seconds after the scan was started. Perfusion parameters including cerebral blood flow (CBF) and cerebral blood volume (CBV) were calculated from the GRE measurement acquired from VSI. If VSI could not be performed, conventional DSC-PWI was performed using a single-shot 2 D Echo Planar Imaging (EPI) sequence (TE = 22 ms, TR = 1880 ms, Flip Angle = 60°, FOV = 230 mm, matrix = 80 × 80, 16 slices, 5-mm slice thickness, no gap, Bandwidth = 1690 Hz/Pixel, GRAPPA Acceleration Factor = 2, 60 time points); the identical dose of 0.13 mL/kg body weight of contrast agent with Gadolinium was injected with a 5 second delay.

### Data processing

All data were managed, annotated, and processed using a local instance of the medical-imaging platform NORA (www.nora-imaging.org, University Medical Center Freiburg). VSI was calculated using a MATLAB-based software package, which was embedded into NORA. Maps of apparent diffusion coefficient (ADC) were calculated from diffusion-weighted images via monoexponential fitting. Data processing consisted of the following steps: Gibbs ringing artifact removal,^19^ co-registration of all image contrasts using SPM8, extraction of the first bolus^20^ and conversion from gradient- and spin-echo (GRE, SE) signals to the corresponding relaxation rates ΔR_2GE_ and ΔR_2SE_, respectively. From these curves, following the approach described previously,^19^ vessel density index (reflected by Q [s^−1/3^]), and vessel size (defined by VS [µm]) were calculated as defined in Kiselev et al.:^1^

$$Q\approx \Delta R_{2SE}/\Delta R_{2GE}^{2/3}$$

$$VS=1.734x{\left(\mathrm{ADC}\;x\;\mathrm{CBV}\right)}^{1/2}/Q^{3/2}$$

The formula requires absolute values for CBV. Since DSC-MRI in general only yields relative CBV (rCBV) values, we constructed pseudo-quantitative but consistent values by scaling rCBV to a literature value of 3.2% in healthy tissue^21^ i.e. the contralateral hemisphere. CBV and CBF were calculated from ΔR_2GE_ following the general tracer-kinetic-approach as previously described in detail.^22^ Here, the post-processing consisted of initial motion correction, bivariate filtering, brain extraction and a deconvolution of the tissue curves with an automatically selected arterial input function (AIF); all automatically selected AIFs were visually checked for anomalies. Due to the software upgrade to VB19 at the MRI scanner, patients scanned between November 2013 and June 2014 (N = 23) could not be measured with combined spin- and gradient-echo, and hence no VSI calculation was possible. For these patients perfusion parameters were calculated from a gradient-echo DSC-PWI.

### Regions of interest selection

**Figure 1.**
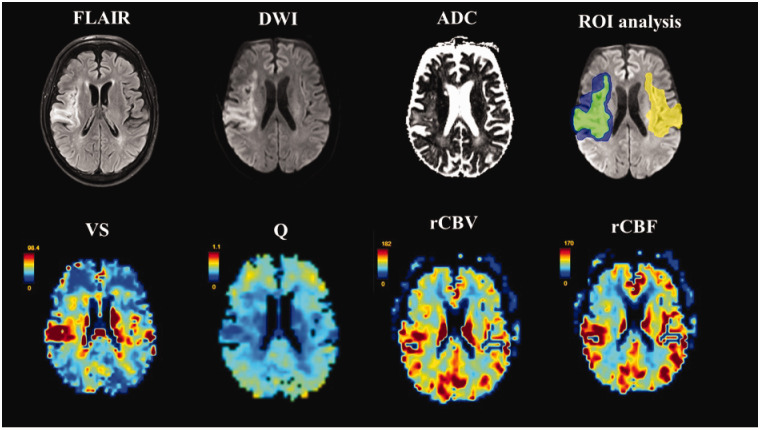
Top row from left to right: FLAIR, diffusion weighted imaging (DWI), and apparent diffusion coefficient (ADC) images with schematic depiction of selected ROI (region of interest) delineations on baseline DWI (green=lesion; blue=perilesional space; yellow=contralateral healthy). Bottom row from left to right: corresponding VS (vessel size; [μm]), Q (vessel density index; [s^−1/3^]), and relative cerebral blood volume (rCBV) and blood flow (rCBF) maps derived from contrast-enhanced vessel size imaging.

The region of interest (ROI) of the ischemic lesion was manually delineated by a single experienced rater (AK) for each patient on DWI with reference to correlating ADC and FLAIR images, while blinded to VSI and perfusion images. The perilesional space ROIs were created via standardized dilation of the ischemic lesion ROI by two voxels in 3 D space with subsequent subtraction of inner ischemic lesion ROI to create a “border zone” for the perilesional space (Figure 1). Furthermore, the ischemic lesion ROI was mirrored to the contralateral healthy side (mirrored lesion).

For all ROIs, CBV and CBF are reported as relative values (rCBV and rCBF = normalized to contralateral hemisphere). Similarly, VS and Q are reported as relative values (rVS and rQ); the supratentorial segment of the contralateral healthy hemisphere was manually delineated for each patient for intra-individual normalization of VS and Q to healthy contralateral tissue (i.e. relative VS expressed as a ratio defined by median VS within lesion/median VS contralateral hemisphere). All derived ROIs (ischemic lesion, perilesional space, and mirrored lesion) were superimposed onto VSI and perfusion maps for evaluation.

### Statistical analysis

Categorical data are presented as absolute numbers and proportions, continuous data as median and interquartile range (IQR) or mean and standard deviation (SD), depending on the respective distribution of data. Normality of distributions of continuous variables was assessed using Shapiro-Wilk test.

In order to assess differences in VSI and perfusion parameters across selected ROIs, we performed four linear mixed-models in which the dependent variables were defined as the MRI parameter of interest (rVS, rQ, rCBV, and rCBF). All models included subjects as a random effect and intervention group, time points (v01 *vs*. v02), as well as time-to-MRI (in days) as fixed effects.

A linear regression analysis was used to assess the association of rVS within the ischemic lesion at v01 and lesion size (assessed on v01 MRI) and stroke severity (assessed at time of acute stroke, and time of v01 and v02 MRI). A univariate and multivariate logistic regression analysis for good outcome (defined as mRS ≤ 2 at six months post stroke) was performed with adjustment for the following parameters (age, rVS within ischemic lesion at v01, stroke severity at time of acute stroke, and lesion volume on v01 MRI). All statistical analyses were performed using STATA (StataCorp 2015 Software release 14).

## Results

### Description of cohort and data

The mean age of the cohort was 68 (SD 11) years, median NIHSS at time of acute stroke was 10 (IQR 6–14). The median time from stroke onset to first MRI (v01) was 27 (IQR 15–34) days. Refer to Table 1 for description of patient demographics.

**Table 1.** Patient demographics and clinical parameters of entire patient cohort.

	All patients (N = 62)
Age in years, mean (SD)	67.9 (10.6)
Male sex, %(n)	56.5 (35)
Cerebrovascular risk factors	
Smoking, %(n)	35.5 (22)
Arterial hypertension, %(n)	79.0 (49)
Diabetes mellitus, %(n)	29.9 (26)
Atrial fibrillation, %(n)	21.0 (13)
Hypolipoproteinemia, %(n)	46.8 (29)
Stroke etiology	
Large-artery atherosclerosis, %(n)	33.9 (21)
Cardioembolism, %(n)	30.7 (19)
Small-vessel occlusion, %(n)	14.5 (9)
Stroke of other determined etiology, %(n)	6.5 (4)
Stroke of undetermined etiology, %(n)	12.9 (8)
NIHSS time of acute stroke, mean (SD)	10 (6–14)
Time from stroke onset to first MRI (v01) in days, median (IQR)	27 (15–34)
Time from onset to second MRI (v02) in days, median (IQR)	61 (47–67)
NIHSS at v01, median (IQR)	6 (3–9)
NIHSS at v02, median (IQR)	4 (2–6)
Wahlund score assessed v01, median (IQR)	5 (3–8)
Lesion volume in mL on v01, median (IQR)	32.2 (9.7–86.4)
Treatment group physical fitness, %(n)	49.2 (30)

SD= standard deviation; IQR=interquartile range; MRI =magnetic resonance imaging; v01 = time point first MRI, v02 = time point second MRI.

Fifty-one patients received both v01 and v02 MRIs. Thirty-one patients had available VSI on v01 MRI and 20 had available VSI on v02 MRI (only 17 had VSI at both v01 and v02). Perfusion maps were available for 53 patients on v01 MRI and 34 patients on v02 MRI. On v01 MRI, mean VS within the ischemic lesion was 56 µm (SD ± 15 µm), mean Q was 0.27 s^−1/3^ (SD ± 0.04 s^−1/3^). For a comprehensive description of quantitative values of VS and Q, as well as rCBF and rCBV on v01 and v02 scan in selected ROIs, refer to Table 2. For a visual depiction of the distribution of rVS, rQ, rCBF and rCBV values across selected ROIs on v01 and v02 scans combined, refer to the violin plots in Supplemental Figure 1. Correlation matrices for all MR variables of interest within the lesion and contralateral healthy ROI are available in Supplemental Figure 2.

**Table 2.** Absolute values of vessel size (VS) and vessel density index (Q) before normalization to contralateral hemisphere, and markers of cerebral perfusion (including relative cerebral blood flow and cerebral blood volume) on first (v01) and second (v02) MRI in selected regions of interest.

	VS (µm)	Q (s^−1/3^)	rCBF (%)	rCBV (%)
	N = 31		N = 53	
v01 MRI				
Lesion	55.9 (±15.1)	0.27 (±0.04)	61.7 (±20.3)	71.1 (±19.2)
Perilesional	50.7 (±12.3)	0.29 (±0.05)	68.5 (±13.6)	81.2 (±16.1)
Mirrored lesion	50.0 (±15.9)	0.30 (±0.06)	86.9 (±19.2)	86.2 (±17.1)
				
	N = 20		N = 34	
v02 MRI				
Lesion	55.8 (±19.9)	0.28 (±0.07)	54.1 (±15.4)	66.5 (±17.9)
Perilesional	47.4 (±14.1)	0.30 (±0.05)	61.7 (±13.6)	78.3 (±16.4)
Mirrored lesion	45.2 (±5.7)	0.31 (±0.03)	86.0 (±25.7)	83.0 (±17.5)

Q=vessel density index, VS=vessel size, CBF=cerebral blood flow, CBV=cerebral blood volume.

Mean ADC within the ischemic lesion was 961 mm^2^/s (SD ± 280 mm^2^/s) and 1135 mm^2^/s (SD ± 353 mm^2^/s) on v01 and v02 MRI, respectively (comprehensive description of ADC values across ROIs at both scanning time-points available in Supplemental Table 2).

### Factors associated with vessel size, density, and perfusion

The results of the linear mixed-models are presented in Table 3. The rVS in the ischemic lesion was higher compared to contralateral mirrored lesion (coefficient 0.21, standard error [SE]±0.08, p = 0.01) and rQ measured in the ischemic lesion was lower than the contralateral mirrored lesion (coefficient −0.10, SE ± 0.03, p < 0.01), following adjustment for repeated and within-subject measurements. There was no difference in rVS nor rQ in the perilesional space compared to the contralateral mirrored lesion. There was no significant association between time-to-MRI (in days) and rVS (coefficient −0.001, SE ± 0.004, p = 0.83) or rQ (coefficient −0.001, SE ± 0.002, p = 0.48).

**Table 3.** Linear mixed-models for each dependent variable (relative vessel size and Q, rCBV, and rCBF).

**Dependent variable: relative vessel size**			
**Fixed-effects**	**Coefficient**	**Std. Error**	**p-value**
Region of interest			
Contralateral healthy	*- reference -*	*- reference -*	–
Perilesional space	0.030	0.080	0.705
Lesion	0.206	0.080	**0.010**
Time to MRI in days	−0.001	0.004	0.831
Time point of MRI (v01 vs. v02)	−0.086	0.140	0.534
Intervention group (training)	−0.136	0.109	0.211
**Random-effects**			
	**Estimate**	**Std. Error**	**95% CI**
Subject ID	0.250	0.048	0.170–0.362
**Dependent variable: relative vessel density**			
**Fixed-effects**			
	**Coefficient**	**Std. Error**	**p-value**
Region of interest			
Contralateral healthy	*- reference -*	*- reference -*	–
Perilesional space	−0.037	0.032	0.255
Lesion	−0.099	0.032	**0.002**
Time to MRI in days	−0.001	0.002	0.477
Time point of MRI (v01 vs. v02)	0.079	0.057	0.165
Intervention group (training)	−0.046	0.045	0.308
**Random-Effects**			
	**Estimate**	**Std. Error**	**95% CI**
Subject ID	0.104	0.020	0.072–0.151
**Dependent variable: relative cerebral blood volume**			
**Fixed-effects**			
	**Coefficient**	**Std. Error**	**p-value**
Region of interest			
Contralateral healthy	*- reference -*	*- reference -*	–
Perilesional space	−6.71	2.68	**0.012**
Lesion	−17.69	2.67	**<0.001**
Time to MRI in days	−0.251	0.128	0.050
Time point of MRI (v01 vs. v02)	2.50	4.26	0.557
Intervention group (training)	−3.87	3.634	0.288
**Random-effects**			
	**Estimate**	**Std. Error**	**95% CI**
Subject ID	11.21	1.59	8.49–14.8
**Dependent variable: relative cerebral blood flow**			
**Fixed-effects**			
	**Coefficient**	**Std. Error**	**p-value**
Region of interest			
Contralateral healthy	*- reference -*	*- reference -*	–
Perilesional space	−20.30	2.83	**<0.001**
Lesion	−27.58	2.81	**<0.001**
Time to MRI in days	−0.248	0.119	**0.037**
Time point of MRI (v01 vs. v02)	4.17	4.07	0.306
Intervention group (training)	−2.20	3.35	0.512
**Random-effects**			
	**Estimate**	**Std. Error**	**95% CI**
Subject ID	9.67	1.49	7.15–13.1

All models included subjects as a random effect and intervention group, time points (v01 vs. v02), as well as time-to-MRI (in days) as fixed effects.

**Figure 2.**
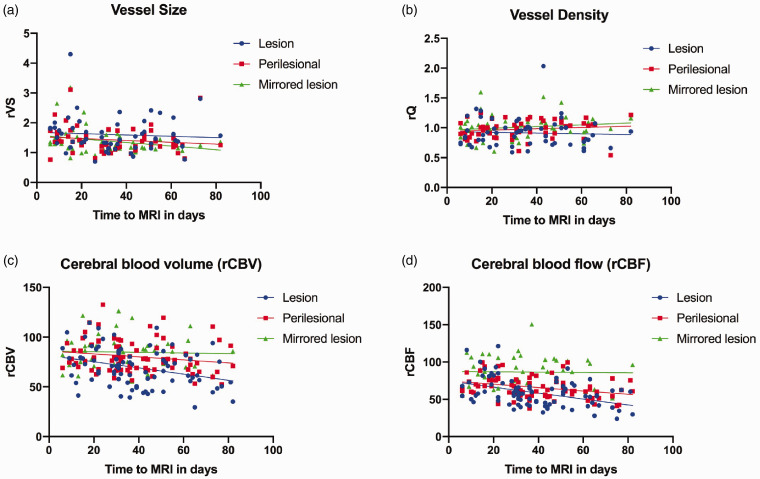
Two-way descriptive scatterplots depicting relative vessel size (rVS), relative vessel density index (rQ), rCBV and cCBF (Panels (a) to (d), respectively) within selected ROIs (ischemic lesion, perilesional space, and contralateral mirrored lesion) based on time to MRI in days. Measurements from v01 and v02 scans are included in all scatter-plots.

Mixed-model analyses for rCBF and rCBV similarly identified the ROI as the main modifying effect; perfusion parameters differed across all selected ROIs including lesion and perilesional space in comparison to the contralateral healthy ROI as a reference. The time-to-MRI was identified as a significant fixed effect for both rCBV and rCBF; perfusion parameters were inversely correlated with time-to-MRI in days (Table 3). Descriptive two-way scatter plots depicting rVS, rQ, and perfusion measurements within selected ROIs based on time-to-MRI in days (v01 and v02 included) are shown in Figure 2.

Patient demographics were balanced between both intervention groups of the PHYS-STROKE trial. The aerobic fitness training intervention group was not identified as a main effect for any of the MRI parameters in the mixed-models (Table 3). Neither VSI, nor perfusion parameters in selected ROIs at v01 and v02, nor absolute changes of VSI and perfusion parameters between v01 and v02 scans differed significantly between intervention groups (for univariate analysis based on intervention group, refer to Supplemental Table 2).

### Associations with stroke severity, lesion size, and functional outcome

Higher rVS values (at v01 MRI) within the ischemic lesion were associated with larger lesion sizes assessed on both v01 MRI (β 34 95% CI 6.2 – 62; p = 0.018) and v02 MRI (β 33 95%CI 5.7 – 60; p = 0.020). Higher VS values within the lesion at v01 were also associated with more severe strokes at the time of symptom onset (β 2.9 95%CI 0.49 – 5.3; p = 0.02), assessed by the NIHSS score. However, there was no association between lesion VS at v01 and NIHSS at the time of v01 MRI (β 0.46 95%CI −1.9 – 2.9, p = 0.70) or v02 MRI (β 0.27 95%CI −1.5 – 2.2, p = 0.68.

Patients who experienced a good outcome (mRS ≤2) six months following enrollment had significantly lower lesion rVS measured on v01 (1.2, IQR 1.0 – 1.5 *vs*. 1.7, IQR 1.3–1.9; p = 0.04) in univariate analysis compared to patients who had a mRS >2. Lesion rQ did not differ between patients who experienced a good *vs*. poor outcome six months post-stroke (1.0, IQR 0.86 – 1.1 vs. 0.85, IQR 0.74 – 0.97; p = 0.28). Multivariate analysis did not identify v01 lesion VS as an independent predictor of functional recovery six months post-stroke (adjusted OR 0.16 95% 0.01 – 5.6, p = 0.32; Table 4).

**Table 4.** Univariate and multivariate regression analysis for good outcome (modified Rankin Score ≤2) six months following enrollment presenting crude and adjusted odds ratios (ORs) with 95% confidence intervals (CI); N = 62.

Binary logistic regression analysis for good outcome (mRS ≤2) at six months					
		Univariate model		Multivariate model	
	Number of cases	Odds ratio (95% CI)	p-value	Odds ratio (95% CI)	p-value
Relative vessel size within lesion assessed on v01 MRI	31	0.14 (0.02–1.3)	0.09	0.16 (0.005–5.6)	0.32
Lesion volume in mL assessed on v01 MRI	57	0.99 (0.97–1.0)	0.05	0.97 (0.93–1.0)	0.14
Stroke severity (NIHSS at v01)	62	0.71 (0.57–0.88)	0.002	0.62 (0.40–0.98)	0.04
Age	62	0.94 (0.89–1.0)	0.03	0.91 (0.81–0.10)	0.12

Among 17 patients with consecutive VSI measurements, changes in rVS and rQ were not associated with lesion progression or outcome at six months after stroke in exploratory analyses (data not shown).

## Discussion

This is the first study to investigate the cerebral microvasculature in patients with subacute ischemic stroke using MRI-based VSI. Linear mixed-models identified an increase in vessel size (VS) with corresponding decrease in vessel density index (Q) within the ischemic lesion compared to corresponding healthy contralateral tissue. There was no difference in VS or Q within the perilesional space compared to the contralateral mirrored ROI, whereas rCBF and rCBV were significantly reduced. Time-to-MRI in days was not associated with VS or Q measured within the selected ROIs in this analysis. Although increased VS within the ischemic lesion was associated with larger lesion volumes and more severe strokes at the time of symptom onset, VS was not predictive of functional outcome at six months after stroke in multivariable analysis.

The findings of this study of patients with subacute ischemic stroke are in line with previous preclinical studies in the acute and subacute phase of stroke^4,5,12^ and one previous clinical study in the acute phase of stroke that also observed increased VS and reduced Q within the ischemic lesion.^6^ The absolute values of previously published Q values in acute stroke patients^7^ are similar to the values of Q reported in this analysis (mean 0.27 s^−1/3^ vs. 0.30 s^−1/3^; Table 2) corroborating the feasibility of this imaging modality in stroke research. In this analysis, VS was calculated as the mean vessel diameter averaged over the capillary population within an imaging voxel. The absolute values of VS within ischemic lesions reported in this analysis are higher compared to those reported previously, (mean 56 μm diameter vs. 18 μm radius).^7^ Although the post-processing pipelines of the VSI sequences were similar, differences in scan resolution and differing patient cohorts analyzed (i.e. differences in stroke severity, lesion size, and time after stroke) may explain this observed difference.

Linear mixed-models identified significant differences in measured rVS and rQ, as well as markers of cerebral perfusion (rCBF and rCBV) across selected ROIs (Table 3). Interestingly, time-to-MRI in days was not identified as a modifying factor of VS or Q values in this analysis. These results may stand in line with a previously published longitudinal VSI study in a murine stroke model, in which early ischemia-induced changes in vascular morphology within and around the ischemic lesion remained constant over time into the subacute phase of stroke.^5^

Although time-to-MRI in days did not modify VSI measurements, time to scan was inversely associated with rCBF and rCBV; in other words these perfusion metrics decreased over time. In the case of large-scale and functional angiogenesis, one might expect to observe an increase in Q with corresponding increases in tissue perfusion. Although previous observations from Boehm-Sturm *et al*. described sparse histological correlates of perilesional angiogenesis four weeks post-stroke in rats, they did not observe MRI correlates in their translational VSI study.^5^ Similarly, in this explorative clinical study, we observed no MRI-based evidence for large-scale changes in vessel size or density within selected ROIs in the subacute phases following ischemic stroke. Still, our observations require validation in larger, longitudinal studies.^23^

Previous pre-clinical studies suggest that increased physical fitness may enhance angiogenesis and influence functional recovery following stroke.^8,24,25^ These served as the foundation for the randomized controlled PHYS-STROKE trial and the accompanying observational biomarker study BAPTISe.^17^ The trial intervention (aerobic training) did not affect VS, Q, or perfusion measurements in linear mixed-model analyses in this cohort. Although this study was not powered to detect differences in absolute VS and Q values between intervention groups, we performed exploratory univariate analyses stratified by treatment allocation (4-weeks of aerobic fitness training *vs*. 4-weeks of relaxation); here, we also did not observe differences in VSI values or relative tissue perfusion within or surrounding the ischemic lesion (Supplemental Table 2).

Interestingly, the enlargement of microvessel diameter within the ischemic lesion measured on v01 MRI was associated with larger lesion volumes and more severe strokes at the time of the index event. There are several hypotheses as to why VS increases within an ischemic lesion: one being that severe cytotoxic edema leads to the compression of small vessels leading to an overall shift in the average vessel size per unit space,^6,7^ and another being that following ischemic tissue damage, failed vascular remodeling leads to the formation of larger, bulky, dysfunctional vessels.^4,8^ The observed decrease in Q is likely explained by widespread cell death following ischemia.^4,26^ Whether the observed increase in VS within the subacute ischemic lesions in this study is directly attributed to the loss of patent capillaries, to abnormal angiogenesis, or both is not entirely clear. Either way based on our results and what we know from preclinical studies, it is reasonable to assume that early large-scale changes in VS within the ischemic lesion are indicative of severe stroke, progression of lesion size, and potentially worse functional recovery. Although larger lesion VS was associated with a worse long-term functional outcome in univariate analysis, multivariate regression analysis did not identify VS within the stroke lesion to be independently associated with outcome (Table 4). However, due to the limited sample size of this study, a larger independent cohort analysis is warranted to investigate the prognostic value of VSI further.

This study has several limitations that should be considered. Firstly, this is a relatively heterogeneous cohort of stroke patients with respect to ischemic lesion pattern (pure subcortical strokes versus territorial strokes with cortical and subcortical affection), stroke etiology, and cerebrovascular risk profiles. However to the best of our knowledge there is no data available on how VS and Q are modified based on ischemic lesion location, cause of stroke, or presence of cerebrovascular risk factors such as long-term diabetes mellitus. These points should be addressed in future studies involving VSI. Furthermore, time from onset to first and second MRI was highly variable. This is due to the inclusion criteria of the randomized-controlled PHYS-STROKE trial. A longitudinal, long-term study with sequential MRIs at predefined time points after stroke should be performed in future analyses for a more precise MR-based quantification of ischemia-induced microvascular changes in the subacute to chronic phases of stroke. In the current analysis relative perfusion values (rCBV and rCBF) were analyzed; a possible limitation of this approach is that the effect of perfusion changes on the contralateral hemisphere cannot be entirely accounted for. Finally, we also observed a relatively high inter-individual variability in terms of absolute VS values assessed within selected ROIs (Table 2; Supplemental Figure 1); previous studies with this sequence have also reported high standard errors within reported VSI meassurements.^5,7^ However, it is important to consider that VSI does not allow for a direct visualization of the cerebrovascular morphology. Values of VSI are based on complex analytical models that allow for an accurate approximation of vessel size and vessel density indices based on a number of theoretical assumptions (i.e. intravascular signal and native blood paramagnetism are neglected).^1^ These assumptions can result in errors up to an order of two,^1,27,28^ however, differences in actual and measured values follow a monotonic function, which preserves the given order of values and hence may even ease detection. In the current study, measured VSI values were normalized against the inter-individual contralateral healthy hemisphere in order to partially compensate for any additional patient individual variations caused by for example age.

Nonetheless, this is the first longitudinal analysis applying this MR-imaging technique to assess the cerebral microvasculature in a well-characterized cohort of subacute stroke patients, and the results support evidence that VSI may aid in the assessment of ischemia-induced changes following moderate to severe subacute stroke in the clinical setting. In comparison to the standard DSC-PWI, VSI has the advantage of having a similar spatial resolution and comparable acquisition time (albeit 2 min longer) while providing the additional valuable information about vessel size and density. Furthermore, VSI may have the advantage of more precisely assessing tissue-at-risk in the acute clinical setting compared to DSC-PWI.^6,29^ However, it is also important to note that there are alternative methods i.e. selected functional MRI (fMRI) sequences that can provide information on vessel size/density as well without the use of a contrast agent. However, the post-processing of fMRI for this purpose is still largely experimental, time-consuming, and therefore not always suitable for routine clinical diagnostics. VSI could therefore have substantial implications in the clinical setting far beyond the scope of ischemic stroke. The role of VSI in predicting stroke recovery however needs to be validated in larger independent cohort analyses.

## Conclusion

In conclusion, the use of VSI as a minimally invasive imaging technique can aid in assessing the in-vivo cerebral microvasculature in the subacute phases following an ischemic stroke. Translating from preclinical studies, we also observed an increase in VS and a decrease in vessel density with corresponding decreased perfusion within the ischemic lesion, compared to contralateral healthy brain. Time from stroke onset to MRI (up to two months post-stroke) did not alter the dynamic of VS or Q. Higher VS values within the ischemic lesion were associated with larger lesion volumes and more severe strokes, but the prognostic role of VSI in terms of long-term functional recovery requires further testing in larger, independent cohorts.

## Supplemental Material

sj-pdf-1-jcb-10.1177_0271678X211010071 - Supplemental material for Magnetic resonance imaging-based changes in vascular morphology and cerebral perfusion in subacute ischemic stroke Click here for additional data file. Supplemental material, sj-pdf-1-jcb-10.1177_0271678X211010071 for Magnetic resonance imaging-based changes in vascular morphology and cerebral perfusion in subacute ischemic stroke by Anna Kufner, Ahmed A Khalil, Ivana Galinovic, Elias Kellner, Ralf Mekle, Torsten Rackoll, Philipp Boehm-Sturm, Jochen B Fiebach, Agnes Flöel, Martin Ebinger, Matthias Endres and Alexander H Nave in Journal of Cerebral Blood Flow & Metabolism
