# Supplementary material for: Magnetic resonance imaging-based changes in vascular morphology and cerebral perfusion in subacute ischemic stroke
Source: J Cereb Blood Flow Metab. 2021 Apr 17;41(10):2617–27. doi: 10.1177/0271678X211010071 (PMC8504415; doi:10.1177/0271678X211010071)
Supplement: sj-pdf-1-jcb-10.1177_0271678X211010071 - Supplemental material for Magnetic resonance imaging-based changes in vascular morphology and cerebral perfusion in subacute ischemic stroke [file sj-pdf-1-jcb-10.1177_0271678X211010071.pdf]

SUPPLEMENTAL DATA

Magnetic resonance imaging-based changes in vascular morphology and cerebral perfusion in subacute ischemic stroke

Anna Kufner, Ahmed A. Khalil, Ivana Galinovic, Elias Kellner, Ralf Mekle, Torsten Rackoll, Philipp Boehm-Sturm, Jochen B. Fiebach, Agnes Flöel, Martin Ebinger, Matthias Endres[*], and Alexander H. Nave[*]

Table of contents

Supplemental files 2

Supplemental Table 1. Main inclusion and exclusion criteria 2

Supplemental Table 2. MRI parameters based on intervention group. 3

Supplemental Figure 1: Violin plots of vessel size, vessel density, and perfusion........ 4

Supplemental Tables

Supplemental Table 1. Summary of main inclusion and exclusion criteria for enrollment in the randomized controlled PYS-STROKE trial and accompanying observational BAPTISe study.

**PHYS-STROKE**	
**Inclusion criteria**<br>- >= 18 years<br>- sub-acute phase of ischemic or haemorrhagic stroke (days 5-45 following symptom onset)<br>- patients were able to sit unsupported for a minimum of 30 seconds<br>- patients were considered able to performe aerobic excerise by responsible trial physician<br>- Barthel Index (BI) <65 at inclusion	**Exclusion criteria**<br>- intracranial haemorrahge from a ruptured aneurysm or AVM<br>- inability to perform physical exercise required from intervention<br>- assisted walking before stroke<br>- severe cardiac or psychiatric co-morbidities
**BAPTISe**	
**Inclusion criteria**<br>- (all inclusion criteria listed for PHYS-STROKE)<br>- ischemic stroke<br>- able to receive at least one MRI before or after intervention	**Exclusion criteria**<br>- stroke due to intracranial hemorrhage<br>- previous subarachnoid hemorrhage or other hemorrhagic stroke<br>- progressive stroke<br>- not able to receive MRI, including perfusion imaging<br>- life expectancy <1 year determined by trial physician<br>- drug or alcohol addiction within the last six months<br>- significant current psychiatric illness defined as medication-refractory (bipolar affective disorder, psychosis, schizophrenia)

Table 2a: Absolute values of ADC (apparent diffusion coefficient) on first (v01) and second (v02) MRI in selected regions of interest.

		ADC [mm²/s] (N=31)
**v01 MRI**	**Lesion**	960.5 (±280.3)
	**Perilesional**	822.2 (±216.9)
	**Mirrored Lesion**	838.6 (±85.1)
		**N=20**
**v02 MRI**	**Lesion**	1135.2 (±353.0)
	**Perilesional**	964.5 (±141.7)
	**Mirrored Lesion**	832.8 (±84.1)

Table 2b: Linear Mixed-model for ADC including subjects as a random effect and intervention group, time points (v01 vs. v02), as well as time-to-MRI (in days) as fixed effects.

**Dependent variable: apparent diffusion coefficent**			
**Fixed-effects**			
	**Coefficient**	**Std. Error**	**p-value**
Region of interest			
Contralateral healthy	*- reference -*	*- reference -*	-
Perilesional space	17.2	37.4	0.646
Lesion	170.9	37.4	**<0.001**
Time to MRI in days	3.9	2.1	**0.056**
Time point of MRI (v01 vs. v02)	-6.9	70.6	0.923
Intervention group (training)	-31.7	54.9	0.563
**Random-effects**			
	**Estimate**	**Std. Error**	**95% CI**
Subject ID	131.8	24.4	91.6 – 189.6

Supplemental Table 3. Comparison of v01 and v02 MRI parameters including lesion growth, assessment of microvasculature quantified via VSI (vessel size and Q) and cerebral perfusion (cerebral blood flow and cerebral blood volume), based on intervention group.

	**Relaxation sessions (N=29)**	**Aerobic fitness training (N=28)**	**p-value**
Lesion volume in mL on v01, median (IQR)	25.6 (9.7 – 85.6)	59.9 (10.6 – 110.1)	0.615
Patterns of ischemic stroke, %(n)			
Territorial	62.1 (18)	53.6 (15)	0.516
Subcortical, supratentorial	37.9 (11)	46.4 (13)	
Stroke severity v01 scan, median (IQR)	4.5 (3 – 7.5)	7.5 (4 – 10)	0.208
Time to v01 MRI in days, median (IQR)	24.5 (14.5 – 37.5)	29 (16 – 32)	0.999
v02 ischemic lesion			
Vessel size	1.4 (1.3 – 1.7)	1.4 (1.2 – 1.6)	0.843
Q	0.93 (0.77 – 1.1)	0.87 (0.74 – 1.0)	0.598
rCBV	74.3 (57.9 – 85.9)	59.6 (54.2 – 77.6)	0.148
rCBF	60.2 (54.2 – 68.4)	51.9 (41.9 – 59.3)	0.129
v02 perilesional space			
Vessel size	1.3 (1.2 – 1.6)	1.2 (1.1 – 1.3)	0.166
Q	1.1 (1.0 – 1.1)	1.0 (0.9 – 1.2)	0.947
rCBV	77.9 (69.1 – 97.1)	70.9 (65.2 – 89.2)	0.117
rCBF	60.7 (56.6 – 75.5)	60.8 (51.4 – 64.4)	0.375
Absolute change ischemic lesion (v02 – v01)	-0.02 (-0.92 – 0.26)	-0.02 (-0.59 – 0.25)	0.939
Vessel size	-0.07 (-0.19 – 0.13)	-0.05 (-0.12 – 0.27)	0.537
Q	-15.5 (-21.6 – -8.21)	-12.1 (-16.7 – -0.64)	0.625
rCBV	-8.4 (19.5 – 0.95)	-8.4 (-19.6 – -1.78)	0.918
rCBF			
Absolute change perilesional space (v02 – v01)	0.17 (-0.32 – 0.27)	-0.29 (-0.56 – 0.05)	0.105
Vessel size	-0.06 (-0.10 – 0.17)	0.11 (-0.01 – 0.21)	0.143
Q	-5.9 (-16.5 – 2.9)	-8.9 (-17.0 – 0.069)	0.603
rCBV	-11.9 (-20.7 – -1.7)	-9.7 (-14.7 – -6.7)	0.664
rCBF			
v02 MRI, %(n)			
- Lost-to follow-up	13.8 (4)	10.7 (3)	0.567
- Hemorrhagic transformation	6.9 (2)	10.7 (3)	
- Silent new lesion	6.9 (2)	10.7 (3)	

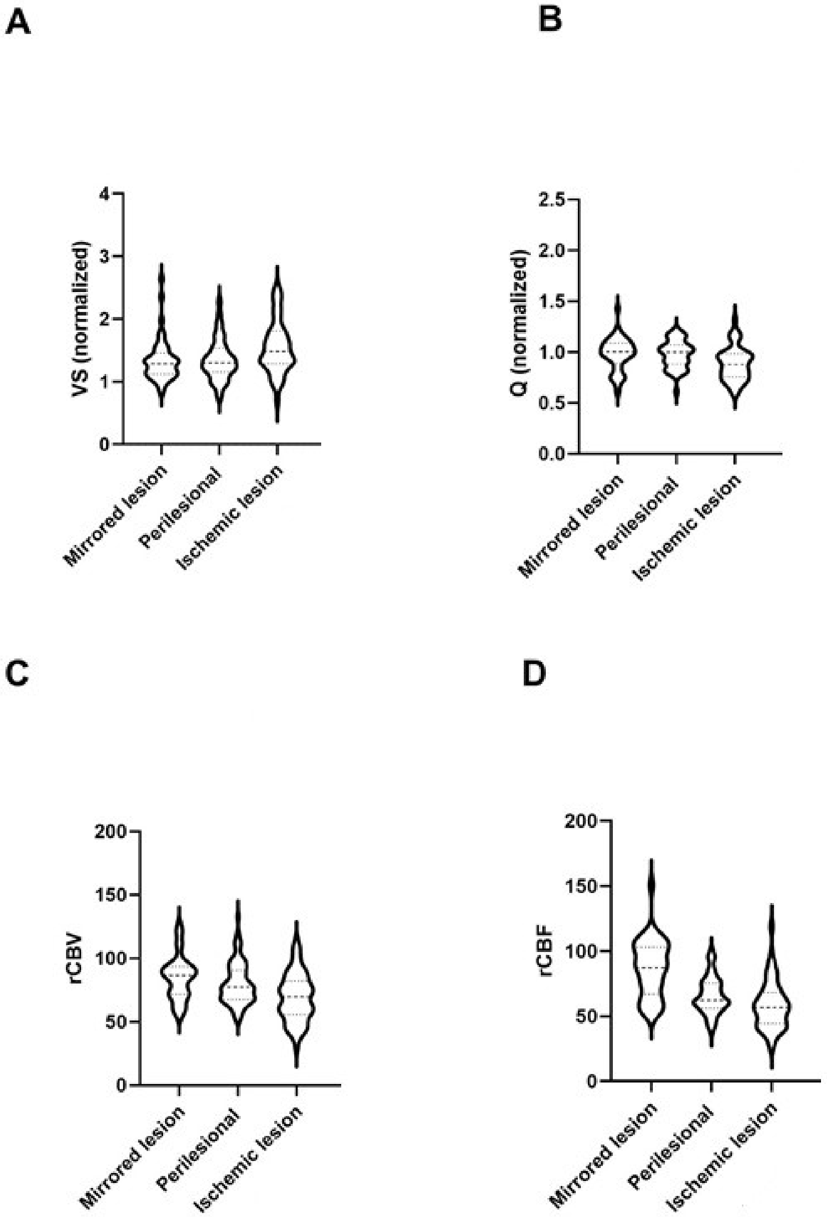

Supplemental Figure 1: Violin plots presenting distribution of normalized A) vessel size (VS) and B) vessel density (Q) and C) relative cerebral blood volume (rCBF) and D) cerebral blood volume (rCBV) in selected regions of interest (ROIs) assessed on v01 and v02 MRI combined (number of patients: n=62; 47 MRIs with VSI, 87 MRIs and perfusion).

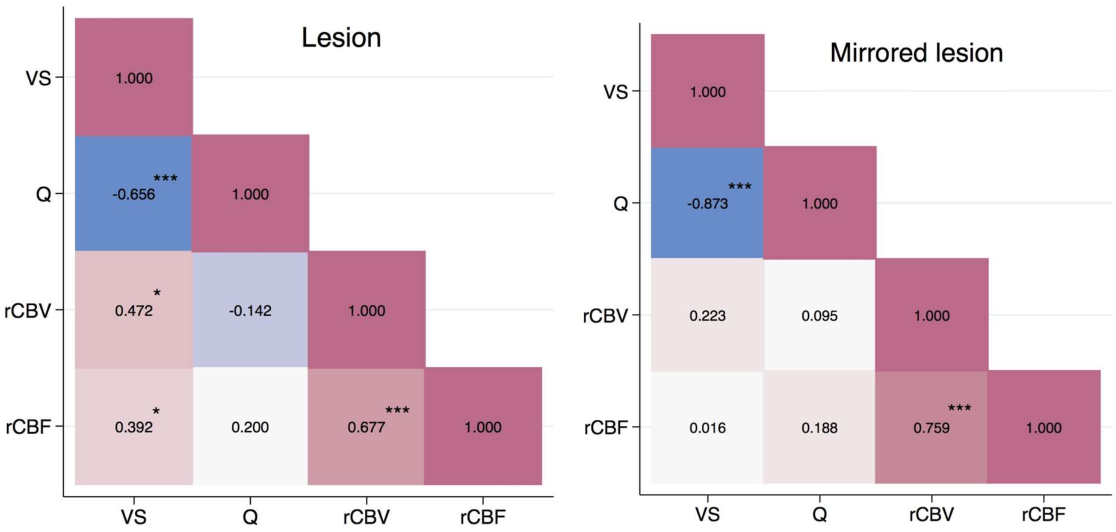

Supplemental Figure 2: Correlation matrices for variables of interest - namely vessel size (VS), vessel density (Q), relative cerebral blood volume (rCBV), and relative cerebral blood flow (rCBF) – within the lesion (left) and contralateral, mirrored lesion (right). *** represents a $p<0.001$, ** represents a $p<0.01$, and * represents a $p<0.05$. Where there is no symbol depicted, the correlation was non-significant.
